# In vitro characterization of pralidoxime transport and acetylcholinesterase reactivation across MDCK cells and stem cell-derived human brain microvascular endothelial cells (BC1-hBMECs)

**DOI:** 10.1186/s12987-016-0035-0

**Published:** 2016-07-11

**Authors:** Erin Gallagher, Il Minn, Janice E. Chambers, Peter C. Searson

**Affiliations:** Institute for Nanobiotechnology Johns Hopkins University, 3400 North Charles Street, Baltimore, MD 21218 USA; Department of Materials Science and Engineering, Johns Hopkins University, 3400 North Charles Street, Baltimore, MD 21218 USA; Department of Radiology and Radiological Science, Johns Hopkins University, 3400 North Charles Street, Baltimore, MD 21231 USA; College of Veterinary Medicine, Mississippi State University, Mississippi State, MS 39762-6100 USA

## Abstract

**Background:**

Current therapies for organophosphate poisoning involve administration of oximes, such as pralidoxime (2-PAM), that reactivate the enzyme acetylcholinesterase. Studies in animal models have shown a low concentration in the brain following systemic injection.

**Methods:**

To assess 2-PAM transport, we studied transwell permeability in three Madin-Darby canine kidney (MDCKII) cell lines and stem cell-derived human brain microvascular endothelial cells (BC1-hBMECs). To determine whether 2-PAM is a substrate for common brain efflux pumps, experiments were performed in the MDCKII-MDR1 cell line, transfected to overexpress the P-gp efflux pump, and the MDCKII-FLuc-ABCG2 cell line, transfected to overexpress the BCRP efflux pump. To determine how transcellular transport influences enzyme reactivation, we developed a modified transwell assay where the inhibited acetylcholinesterase enzyme, substrate, and reporter are introduced into the basolateral chamber. Enzymatic activity was inhibited using paraoxon and parathion.

**Results:**

The permeability of 2-PAM is about 2 × 10^−6^ cm s^−1^ in MDCK cells and about 1 × 10^−6^ cm s^−1^ in BC1-hBMECs. Permeability is not influenced by pre-treatment with atropine. In addition, 2-PAM is not a substrate for the P-gp or BCRP efflux pumps.

**Conclusions:**

The low permeability explains poor brain penetration of 2-PAM and therefore the slow enzyme reactivation. This elucidates one of the reasons for the necessity of sustained intravascular (IV) infusion in response to organophosphate poisoning.

**Electronic supplementary material:**

The online version of this article (doi:10.1186/s12987-016-0035-0) contains supplementary material, which is available to authorized users.

## Background

The blood–brain barrier (BBB) is a dynamic system responsible for maintaining homeostasis by regulating the chemical environment, immune cell transport, and the entry of toxins into the CNS [1–3]. Neurotoxins are microorganisms, viruses, bacterial toxins, and chemicals that disrupt neurological function [2, 4]. Organophosphates (OPs) are a class of chemical neurotoxicants comprised of a central phosphate surrounded by electronegative atoms, such as oxygen and sulfur. While widely used as insecticides, nerve agents such as sarin and VX, are also organophosphates [5]. In the brain and body, organophosphates persistently bind to the active site of acetylcholinesterase, blocking breakdown of the neurotransmitter acetylcholine [6, 7].

Organophosphate poisoning is usually treated with oximes, such as pralidoxime (2-PAM), that reactivate acetylcholinesterase [7]. The FDA protocol for organophosphate poisoning involves immediate intramuscular (IM) injection followed by intravascular (IV) administration [6, 8]. IM injection of 2-PAM is usually co-administered with atropine and/or diazepam. Typical IV dosing of 2-PAM, depending on symptoms and exposure pathway, involves administration of 1 g in 100 mL^−1^ saline over 15–30 min followed by continuous infusion of 500 mg h^−1^ (about 700 µM in blood) [6, 7].

2-PAM is an ionic molecule and hence the permeability across the blood–brain barrier has been assumed to be very low [9, 10]. Clinical trials have shown that 2-PAM is rapidly cleared from the body [11, 12], highlighting the need for continuous infusion to maintain a therapeutic dose [11, 13–15]. Based on animal studies, the minimum effective concentration in blood is reported to be around 4 mg L^−1^ (about 30 µM) [9, 15].

Therefore, to assess transport into the brain we studied permeability of 2-PAM in four cell lines: MDCKII, MDCKII-MDR1, MDCKII-FLuc-ABCG2, and BC1-hBMECs. Madin-Darby canine kidney epithelial cells (MDCKs) are widely used for in vitro assessment of brain penetration and the permeability values for a wide range of solutes have been reported [16]. The MDCKII-MDR1 cell line is transfected to express the human P-gp efflux pump, and the MDCKII-FLuc-ABCG2 line is transfected to overexpress BCRP efflux pump. Human brain microvascular endothelial cells (BC1-hBMECs) are derived from human-induced pluripotent stem cells (hiPSCs) [17–19].

## Materials

### Cell lines

MDCKII and MDCKII-MDR1 cells were obtained from the Netherlands Cancer Institute (NKI) [20]. Following NKI’s protocol, cells were cultured in DMEM (High Glucose, GlutaMAX) with 10 % Fetal Bovine Serum (FBS, ATCC, Manassas, VA, USA), and 1 % penicillin–streptomycin (ATCC). MDCKII/ABCG2 cells expressing ABCG2/BCRP were provided by the Pomper Group (JHU). MDCKII cells were transfected with pGL4.16 [luc2cp/Hygro] (Promega, Madison, WI, USA) [21, 22]. Cells were maintained in MEM-l-glutamine (Life Tech, Carlsbad, CA, USA), 10 % FBS HI, 1 mg mL^−1^ G418 (Geneticin, Life Tech), and 100 µg mL^−1^ Hygromycin B (Life Tech) [22].

Human brain microvascular endothelial cells (BC1-hBMECs) were derived from iPSCs, based on a protocol reported by Lippmann et al. [17, 18]. Briefly, BC1 iPSCs [23] were cultured for 4 days in TeSR-E8 Basal Medium (05940, Stem Cell Technologies, Vancouver, BC, Canada) on growth factor-reduced Matrigel, 40 µg mL^−1^ (354230, Fisher Scientific, Pittsburgh, PA, USA) [19]. Once cells formed substantial colonies, they were placed in UM/F- unconditioned media (DMEM/F12, 20 % KOSR, 0.5 % l-glutamine, 1 % NEAA, 0.836 µM Beta-Mercaptoethanol) for 6 days. For the final 2 days of differentiation, the cells were placed in endothelial cell serum-free media (EC, 1 % human platelet poor-derived serum, 20 ng mL^−1^ bFGF) with 10 µM all trans retinoic acid (Sigma, St. Louis, MO, USA) to promote preferential growth of the BC1-hBMECs. The cells were then sub-cultured onto the transwell supports coated with collagen IV (100 μg mL^−1^; Sigma) and fibronectin (50 μg mL^−1^; Sigma) and cultured for two more days prior to performing permeability experiments [19]. Characterization of the BC1-hBMEC cells has been reported elsewhere [19].

### Permeability measurements

#### 2-PAM

MDCK cells were seeded on transwells (24 well; PE; 0.33 cm^2^ area; 0.4 µm pore diameter, Corning, Corning, NY, USA) coated with collagen I (rat tail, 10 µg cm^−2^, Corning) at a density of 6 × 10^5^ cells cm^−2^. Permeability experiments were performed 4 days after seeding. The medium was changed 2 h before the experiment and transepithelial electrical resistance (TEER) was measured before all experiments (Endohm/EVOM2). For MDCK cells, permeability measurements were performed if the TEER was ≥90 Ω cm^2^ (see Additional file 1: Table S1: TEER values for transwell experiments) [24, 25]. To confirm the integrity of the MDCK monolayers, the permeability of Lucifer yellow, 100 µM, was measured for up to 2 h. In all cases the permeability was ≤1 × 10^−6^ cm s^−1^: 0.71 ± 0.34 × 10^−6^ cm s^−1^ (MDCKII), 0.38 ± 0.20 × 10^−6^ cm s^−1^ (MDCKII-MDR1), and 0.46 ± 0.21 × 10^−6^ cm s^−1^ (MDCKII-FLuc-ABCG2). The measured permeability for Lucifer yellow under the same conditions in transwells without cells, was 4.35 × 10^−5^ cm s^−1^.

All experiments with MDCK cells were performed in Hank’s balanced salt solution (HBSS) with 10 mM HEPES (Sigma) and 15 mM glucose (Sigma), pH 7.4. After incubation in media for 2 h, cell monolayers were immersed in fresh HBSS for 30 min to remove any traces of media. Then 100 µM or 10 µM 2-PAM (pralidoxime chloride, Sigma) in HBSS was pipetted into either the apical or basolateral chamber, with HBSS on the receiving side. Cell monolayers with test solutes were incubated at 37 °C with 5 % CO_2_ on a rocker to ensure good mixing.

For experiments with BC1-hBMECs, cells were seeded on transwells (24 well; PE; 0.33 cm^2^ area; 0.4 µm pore diameter, Corning) coated overnight with a 50/50 mixture collagen IV (100 μg mL^−1^; Sigma) and fibronectin (50 μg mL^−1^; Sigma) at a density of 1 × 10^6^ cells mL^−1^. For BC1-hBMECs, permeability measurements were performed with cells that had a TEER of  ≥1500 Ω cm^2^. Experiments were performed in transport buffer (distilled Millipore water with 0.12 M NaCl, 25 mM NaHCO_3_, 3 mM KCl, 2 mM MgSO_4_, 2 mM CaCl_2_, 0.4 mM K_2_HPO_4_, 1 mM HEPES, and 0.1 % human platelet poor-derived serum) without the pre-incubation of media or rocking of the cells. After 2 days in EC media, 100 µM of 2-PAM in transport buffer was pipetted into the apical or basolateral well with transport buffer on the receiving side.

The concentration of 2-PAM was determined by HPLC (1260 Infinity HPLC, Agilent, Santa Clara, CA, USA) with UV Vis detection at 296 nm. Solvents were degassed by sonication for 45 min before use and all samples were run at room temperature. An isocratic flow of 44 vol.% acetonitrile (HPLC grade, Chromasolv, Sigma) and 56 vol.% ammonium acetate (0.03 M; HPLC grade, Sigma), pH 4.5, was used with a PolyCAT A column (100 × 2.1 mm, 5 µM, 300 Å, 102CT05-03, Poly LC Inc, Columbia, MD, USA) [26]. Calibration curves were constructed from standard solutions with concentrations of 0.1, 1, 10 and 100 µM. Due to the simplicity of the procedure, no internal standard was used.

#### 2-PAM/atropine

To assess whether atropine, which is often co-administered with 2-PAM, modulates the transport of 2-PAM we performed experiments where MDCK cells were pre-treated with atropine. After 2 h incubation in media, and 30 min rinse in HBSS, MDCKII monolayers were pre-treated with 1 µM atropine for 30 min, rinsed in HBSS for 5 min, and then incubated in 100 µM 2-PAM for permeability measurements using the same procedure as described above. 2-PAM concentrations were measured by HPLC with an isocratic flow of 55 vol.% acetonitrile and 45 vol.% ammonium acetate (0.03 M).

#### Rhodamine 123

To confirm the up-regulation and polarization of P-gp efflux pumps to the apical face, we measured the permeability of Rhodamine 123, a known P-gp substrate, across MDCKII and MDCKII. MDR1 monolayers [27]. Permeability experiments were performed for 60 min at a concentration of 50 µM. The concentration of Rhodamine 123 (excitation 486 and emission 523) was measured by fluorescence (Fluorolog, Horiba Scientific, Edison, NJ, USA). Calibration curves were generated over the concentration range from 0.001 to 1 µM.

### Coupled permeability and acetylcholinesterase reactivation

To assess coupled 2-PAM transport and enzyme reactivation, experiments were performed in a transwell device with acetylcholinesterase in the basolateral chamber. Confluent monolayers of MDCKII cells were formed as described above. Electric eel acetylcholinesterase (AChE, 1U or about 1 µL, >1000 U/mg, Sigma) was placed into the basolateral chamber (24 well plate). A mixture of Ellman’s reagent (DTNB), final concentration 300 µM, and acetylthiocholine (ASCh), final concentration 450 µM, dissolved in HBSS, was introduced into the basolateral chamber, to give a final volume of 600 µL. The time-dependent activity of the enzyme was determined from the absorbance of the DTNB reporter at 412 nm using a plate reader (Spectramax M3). Results were normalized to the activity of the uninhibited enzyme, un-normalized data are provided in the Additional file 1 (Figure S1: Non-normalized reactivation data).

Inhibition was achieved by incubating the enzyme with 0.72 mM parathion (PESTANAL-grade, Sigma) or 4.6 µM paraoxon (PESTANAL-grade, Sigma) for 20 min prior to experiments. In inhibition experiments, the enzyme was inhibited with parathion. Paraoxon, a metabolite of parathion, is about three orders of magnitude more potent as an anticholinesterase inhibitor [28]. The parathion concentration was 157-fold higher than the paraoxon concentration, reflecting their different activities. For reactivation experiments, 2-PAM was introduced into either the apical or basolateral chamber in HBSS. Introducing 2-PAM into the basolateral chamber simulates reactivation alone, whereas introduction of 2-PAM into the apical chamber simulates coupled trans-endothelial transport and reactivation.

#### Positive control (uninhibited enzyme + substrate)

To assess the kinetics of enzyme interaction with the substrate, uninhibited enzyme (AChE), along with substrate (ASCh), and reporter (DTNB) in HBSS were introduced into the basolateral chamber. An apical transwell chamber with a monolayer of MDCK cells was located on the top of the basolateral chamber to ensure that the control was performed in the same way as the other experiments.

#### Negative control (inhibited enzyme + substrate)

To assess the efficiency of enzyme inhibition, AChE was mixed for 20 min with concentrated parathion (0.72 mM final concentration) or paraoxon (4.6 µM final concentration) organophosphates (OP). The inhibited enzyme (AChE-OP) was then placed in the basolateral chamber with substrate (ASCh) and reporter (DTNB) in HBSS. An apical transwell chamber was located on the top of the basolateral chamber as described above.

#### Direct interaction of 2-PAM (inhibited enzyme + substrate + reactivator)

To assess the kinetics of direct reactivation of inhibited enzyme, 100 µM 2-PAM was introduced in the basolateral chamber with the inhibited enzyme (AChE-OP), substrate (ASCh) and reporter (DTNB) in HBSS. An apical transwell chamber was located on the top of the basolateral chamber as described above.

#### Coupled transport of 2-PAM and reactivation

To evaluate the coupled transport of 2-PAM across a cell monolayer and reactivation of inhibited enzyme, 100 µM of 2-PAM was introduced into the apical chamber, with inhibited enzyme (AChE-OP), reporter (DTNB), and substrate (ASCH) in the basolateral chamber.

#### Statistics

Permeability, activity, and reactivation half-time represent the mean ± standard deviation. Statistical significance was determined using a student’s *t* test (two-tailed with unequal variance) with *p* < 0.01 ** and *p* < 0.001 ***. The average permeability values for the MDCK cell lines were calculated from analysis of all of the replicates. Due to variations between differentiations, the average permeability across the BC1-hBMECs was calculated from the average values from each differentiation. Similarly, the efflux ratio was calculated from the average value obtained from each differentiation.

## Results

### Permeability of 2-PAM

**Fig. 1:**
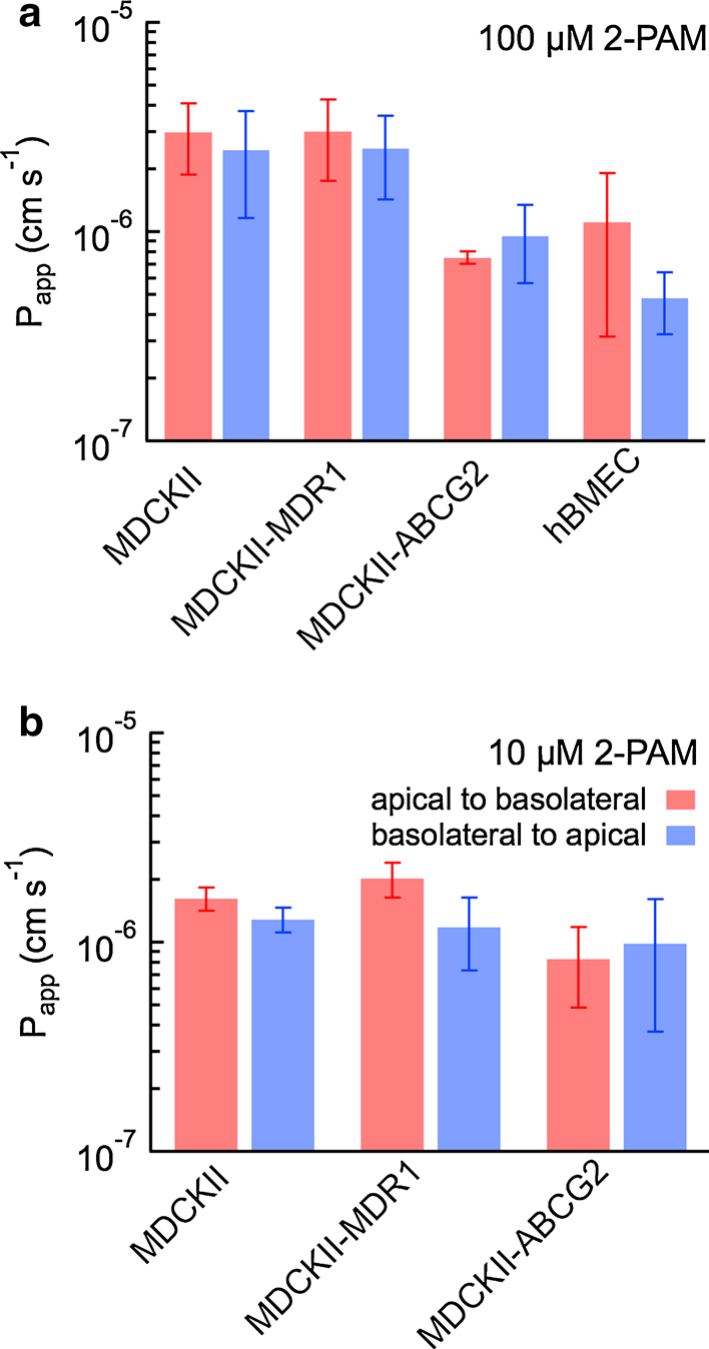
**a** 2-PAM permeability in four different cells lines at a concentration of 100 µM. **b** 2-PAM permeability of in three different cells lines at a concentration of 10 µM. Basolateral-to-apical (*red*), basolateral-to-apical (*blue*). Permeability is reported as mean ± standard deviation. Experiments were performed in HBSS (MDCKII, MDCKII-MDR1, MDCKII-ABCG2) or transport buffer (BC1-hBMEC)

To assess the transport of 2-PAM and to determine whether 2-PAM is an efflux pump substrate, transwell experiments were performed in three cell lines: MDCKII, MDCKII-MDR1, MDCKII-FLuc-ABCG2 at concentrations of 10 and 100 µM (Table 1; Fig. 1). In 10 µM 2-PAM, the average apical-to-basolateral permeability in MDCKII and MDCKII-MDR1 monolayers was about 2 × 10^−6^ cm s^−1^. The average permeability of MDCKII-ABCG2 monolayers was slightly lower, 0.83 × 10^−6^ cm s^−1^, although the difference compared to the MDCKII and MDCKII-MDR1 monolayers was not significant. For 100 µM 2-PAM, the average permeability in MDCKII and MDCKII-MDR1 monolayers increased to about 3 × 10^−6^ cm s^−1^; this increase was significant in MDCKII cells (*p* = 0.05), but not significant in MDCKII-MDR1 cells. The average permeability in MDCKII-ABCG2 cells was 0.76 × 10^−6^ cm s^−1^, very close to the value in 10 µM 2-PAM. The average basolateral-to-apical permeability was very close to the apical-to-basolateral value in all three cell lines with no statistical difference. The apical-to-basolateral permeability of 2-PAM across the stem-cell derived BC1-hBMECs was 1.12 ± 0.80 × 10^−6^ cm s^−1^, comparable to the permeability across the MDCKII cells. The basolateral-to-apical permeability (0.49 ± 0.16 × 10^−6^ cm s^−1^) was lower (*p* = 0.05).

**Table 1:** Permeability of pralidoxime (2-PAM), rhodamine 123 R123) and Lucifer yellow (Ly) across MDCKII, MDCKII-MDR1, MDCKII-FLuc-ABCG2, and BC1-hBMEC monolayers

	P_app_ A→B (cm s^−1^)	N	P_app_ B→A (cm s^−1^)	N	Efflux ratio
*100* *µM 2-PAM*					
MDCKII	2.99 ± 1.12 × 10^−6^	11	2.48 ± 1.30 × 10^−6^	8	0.82
MDCKII-MDR1	3.01 ± 1.27 × 10^−6^	8	2.51 ± 1.08 × 10^−6^	7	0.83
MDCKII-FLuc-ABCG2	0.76 ± 0.05 × 10^−6^	7	0.98 ± 0.40 × 10^−6^	7	1.30
BC1-hBMECs	1.12 ± 0.80 × 10^−6^ (n = 5)	18	0.49 ± 0.16 × 10^−6^ (n = 3)	12	0.84
*10* *µM 2-PAM*					
MDCKII	1.62 ± 0.21 × 10^−6^	6	1.29 ± 1.76 × 10^−6^	7	0.80
MDCKII-MDR1	2.03 ± 0.14 × 10^−6^	8	1.18 ± 0.45 × 10^−6^	8	0.58
MDCKII-FLuc-ABCG2	0.83 ± 0.35 × 10^−6^	7	0.99 ± 0.62 × 10^−6^	7	1.18
*50* *µM R123*					
MDCKII	0.30 ± 0.20 × 10^−6^	6	3.18 ± 0.60 × 10^−6^	5	10.7
MDCKII-MDR1	0.21 ± 0.21 × 10^−6^	12	4.36 ± 0.41 × 10^−6^	11	20.3
*100* *µM LY*					
MDCKII	0.71 ± 0.34 × 10^−6^	7			
MDCKII-MDR1	0.38 ± 0.20 × 10^−6^	7			
MDCKII-FLuc-ABCG2	0.46 ± 0.21 × 10^−6^	8			
*2-PAM atropine*					
MDCKII	2.54 ± 0.33 × 10^−6^	7			

A→B represents apical-to-basolateral permeability, and B→A represents basolateral-to-apical permeability. Permeability values are reported as mean ± standard deviation. The efflux ratio is the ratio of basolateral-to-apical permeability divided by the apical-to-basolateral permeability. For MDCK cells, permeabilities and efflux ratios were calculated from the total number of replicates (N). Data were obtained from at least three independent experiments each with two or more replicates. For the BC1-hBMECs, the permeabilities and efflux ratios were calculated from the average of each differentiation, where N represents the number of independent differentiations

### Influence of atropine on 2-PAM permeability

To determine whether atropine, co-administered with 2-PAM, modulates the permeability of 2-PAM, we measured the permeability of 2-PAM following pre-treatment of the MDCKII monolayer with atropine. The permeability of 2-PAM was 2.54 ± 0.33 × 10^−6^ cm s^−1^, which was not significantly different to the value of 2.99 ± 1.12 × 10^−6^ cm s^−1^ obtained without pre-treatment with atropine (Table 1).

### Permeability of rhodamine 123

To confirm the polarization of the P-gp efflux pumps to the apical surface of the MDCK cells, we measured the permeability of 50 µM rhodamine 123, a known substrate for the P-gp efflux pump [27, 29], in MDCKII and MDCKII-MDR1 cells (Table 1). The basolateral-to-apical permeability of rhodamine 123 in MDCKII was 3.18 × 10^−6^ cm s^−1^, with an apical-to-basolateral permeability of 0.30 × 10^−6^ cm s^−1^ (*p* = 0.001), corresponding to an efflux ratio of 10.7. For the MDCKII-MDR1 cells, the basolateral-to-apical permeability was 4.36 × 10^−6^ cm s^−1^, with an apical-to-basolateral permeability of 0.22 × 10^−6^ cm s^−1^ (*p* = 0.001) corresponding to an efflux ratio of 20.3. The basolateral-to-apical permeabilities to rhodamine in MDCKII and MDCKII. MDR1 cells were significantly different (*p* = 0.02) supporting upregulation and polarization of P-gp efflux pumps to the apical side of the MDCKII.MDR1 cells. Apical-to-basolateral permeabilities of 0.83 × 10^−6^ and 0.89 × 10^−6^ cm s^−1^, with corresponding efflux ratios of 9 and 115, have been reported for transport of 5 µM rhodamine 123 across MDCKII.MDR1 cells [27, 29]. The reported efflux ratio for rhodamine in BC1-hBMEC cells is approximately 4 [19].

### Coupled transport and acetylcholinesterase (AChE) reactivation

**Fig. 2:**
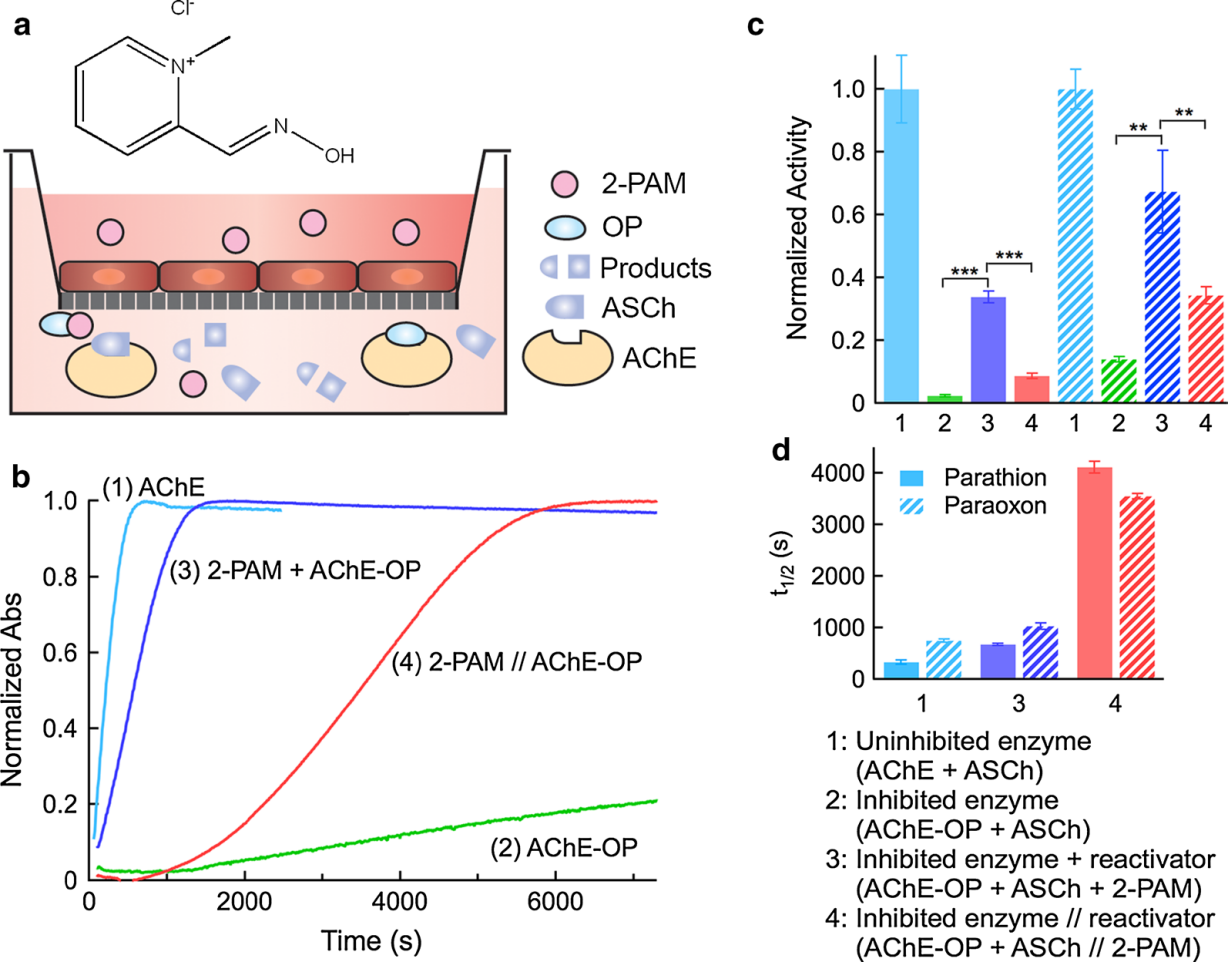
**a** Schematic illustration of the modified transwell assay for measurement of coupled transcellular transport and enzymatic activity, and the chemical structure of 2-PAM. **b** Representative normalized absorbance versus time plots for reactivation of AChE. *1* AChE + ASCh: positive control (uninhibited enzyme + substrate). *2* AChE-OP + ASCh: negative control (inhibited enzyme + substrate). *3* AChE-OP + ASCh + 2-PAM: reactivation with no transport (inhibited enzyme + substrate + reactivator). *4* 2-PAM//AChE-OP + ASCh: transcellular transport + reactivation. **c** Normalized AChE activity (dA/dt) obtained from absorbance versus time curves at the inflection point. The legend provides the details of each experiment. **d** Half-time for AChE reactivation. Data represent mean ± SD. All reactivation experiments were performed in HBSS

To assess the coupled transcellular transport and AChE reactivation, we performed transwell experiments with a monolayer of MDCKII cells and inhibited enzyme in the basolateral chamber (Fig. 2a). Acetylcholinesterase was inhibited with an organophosphate (parathion or paraoxon) for 20 min and then introduced into the basolateral chamber of a transwell device, along with acetylthiocholine (ASCh) and the colorimetric reporter (DTNB). Control experiments were performed to confirm the activity of the enzyme and effectiveness of the inhibitor.

In the absence of 2-PAM, the activity of the inhibited enzyme (AChE-OP) increased very slowly during the 2 h experiment (Fig. 2b). When 2-PAM was introduced into the basolateral chamber with inhibited enzyme, reactivation occurred much more quickly (Parathion: inhibited enzyme and direct reactivation *p* < 0.001, Paraoxon: inhibited enzyme and direct reactivation *p* < 0.01). However, when 2-PAM was introduced into the apical chamber, reactivation of the inhibited enzyme was slowed considerably due to the coupled transport and reactivation (Parathion: direct reactivation and transport reactivation *p* < 0.001, paraoxon: direct reactivation and transport reactivation *p* < 0.01).

The activity of the enzyme following transcellular transport of 2-PAM across MDCKII monolayers (3.49 × 10^−4^ abs s^−1^) was about four fold lower than when the inhibited enzyme was directly exposed to 2-PAM (1.35 × 10^−3^ abs s^−1^) (Fig. 2c). Similarly, the half-time for reactivation of the substrate increased sixfold from 680 s for direct reactivation to 4100 s following transcellular transport (Fig. 2d).

## Discussion

### Transendothelial transport

The permeability of 2-PAM was between 1 × 10^−6^ and 2 × 10^−6^ cm s^−1^ in all three MDCK cell lines, lower than for most central nervous system drugs, which typically have permeabilities greater than 1 × 10^−5^ cm s^−1^ [30]. However several CNS drugs have permeabilities similar to 2-PAM, including the antipsychotics perphenazine (*p* = 1.8 × 10^−6^ cm s^−1^) and fluphenazine (*p* = 3.5 × 10^−6^ cm s^−1^), the anti-anxiety drug sertralin (Zoloft) (*p* = 2.1 × 10^−6^ cm s^−1^), and the analgesic, morphine (*p* = 2 × 10^−6^ cm s^−1^) [16]. While morphine has a low permeability in MDCK cells, the therapeutic dose is particularly low [16]. The low permeability of 2-PAM explains the reported low concentration in the brain in animal studies [10] and the recommended sustained clinical infusion in a clinical setting [6].

MDCK cells are widely used to assess brain penetration of small molecules. Although MDCK cell lines are epithelial in origin and not human, they express tight junction proteins, which limit paracellular transport. Variants such as MDCKII-MDR1 can be used to determine whether a solute is an efflux pump substrate. The stem cell derived BC1-hBMECs exhibit high transendothelial electrical resistance (TEER > 1000 Ω cm^2^), low permeability to solutes such as Lucifer yellow, and express tight junction proteins (e.g. claudin-5), transporters (e.g. LAT-1), and efflux pumps (e.g. P-gp) [17, 19]. The permeability of the stem cell derived BC1-hBMECs (*p* = 1.12 ± 0.80 × 10^−6^ cm s^−1^) was slightly lower than values obtained in MDCK cells, but in the range that is consistent with slow accumulation in the brain.

High permeability values are usually associated with small molecular weight and moderate lipophilicity [31, 32]. While 2-PAM has a molecular weight under 500 Da (172 Da), fewer than 5 hydrogen bond donors (1), and fewer than 10 hydrogen bond acceptors (2), the charge results in a low lipophilicity and hence 2-PAM is not expected to have a high permeability.

There was no significant difference between apical-and-basolateral and basolateral-to-apical permeabilities in MDCK cells, indicating that 2-PAM is not a substrate of the P-gp or ABCG2 pumps. To confirm the polarized expression and activity of the P-gp efflux pumps, we determined efflux ratios of 10.7 and 20.3 for the MDCKII and MDCKII. MDR1 cell lines for the known P-gp substrate rhodamine 123.

Treatment for organophosphate poisoning involves co-administration of 2-PAM and atropine. The permeability of 2-PAM was the same in MDCKII cells and cells pretreated with atropine, showing that atropine does not modulate the permeability of 2-PAM.

### Coupled transcellular transport and enzyme reactivation

To study coupled transcellular transport of the neurotoxin antidote 2-PAM with enzyme reactivation, we developed a modified transwell assay with inhibited enzyme (AChE-OP), substrate (ASCh), and reporter (DTNB) in the basolateral chamber. When 2-PAM was introduced into the apical chamber of the transwell device, the activity of the enzyme decreased four fold compared to the case where 2-PAM was introduced directly into the basolateral chamber. Similarly, the half-time for reactivation of the enzyme increased six fold when coupled to transcellular transport. These results highlight the difficulty in maintaining a therapeutic dose when the permeability is low.

## Conclusions

The permeability of the nerve agent reactivator 2-PAM is 1 × 10^−6 ^– 2 × 10^−6^ cm s^−1^ and is not influenced by pre-treatment with atropine. In addition, 2-PAM is not a substrate for the P-gp or BCRP/ABCG2 efflux pumps. Similar permeability values were obtained for human brain microvascular endothelial cells derived from induced pluripotent stem cells. In a modified transwell assay to couple transcellular transport and enzyme reactivation, we showed that transcellular transport decreased enzymatic activity four fold and increased the reactivation half-time six fold. The low permeability explains poor brain penetration of 2-PAM and the necessity for sustained IV infusion in response to organophosphate poisoning.

## Additional file

10.1186/s12987-016-0035-0 Table S1.

## Abbreviations

MDCK: Madin-Darby canine kidney epithelial cells
2-PAM: pralidoxime chloride
BC1-hBMEC: stem cell derived human brain microvascular endothelial cells
TEER: transendothelial electrical resistance
MDR1: multi-drug resistance gene
P-gp: P-glycoprotein
ABCG2: ATP-binding cassette sub-family G member 2
BCRP: breast cancer resistance protein
ASCh: acetylthiocholine
AChE: acetylcholinesterase
AChE-OP: inhibited enzyme
DTNB: 5,5′-dithiobis-(2-nitrobenzoic acid) (Ellman’s reagent)
