# Supplementary material for: In vitro characterization of pralidoxime transport and acetylcholinesterase reactivation across MDCK cells and stem cell-derived human brain microvascular endothelial cells (BC1-hBMECs)
Source: Fluids Barriers CNS. 2016 Jul 11;13:10. doi: 10.1186/s12987-016-0035-0 (PMC4939658; doi:10.1186/s12987-016-0035-0)
Supplement: Supplementary file 1 — 10.1186/s12987-016-0035-0 Table S1. [file 12987_2016_35_MOESM1_ESM.docx]

# In vitro characterization of pralidoxime transport and acetylcholinesterase reactivation across MDCK cells and stem cell derived human brain microvascular endothelial cells (BC1-hBMECs)

Erin Gallagher,^1,2^ Il Minn,^3^ Janice E. Chambers,^4^ and Peter C. Searson^1,2^*

**1. TEER values for transwell experiments**

| **Cell Line** | **Average TEER (Ω cm^2^)** | **N (number of wells)** |
| --- | --- | --- |
| MDCKII | 109 ± 18 | 57 |
| MDCKII-MDR1 | 129 ± 20 | 61 |
| MDCKII-FLuc-ABCG2 | 583 ± 67 | 36 |
| BC1-hBMECs | 3887 ± 497 | 30 |

**Table S1**. TEER values for all experiments. TEER is reported as average ± standard deviation

**2. Non-normalized reactivation data**


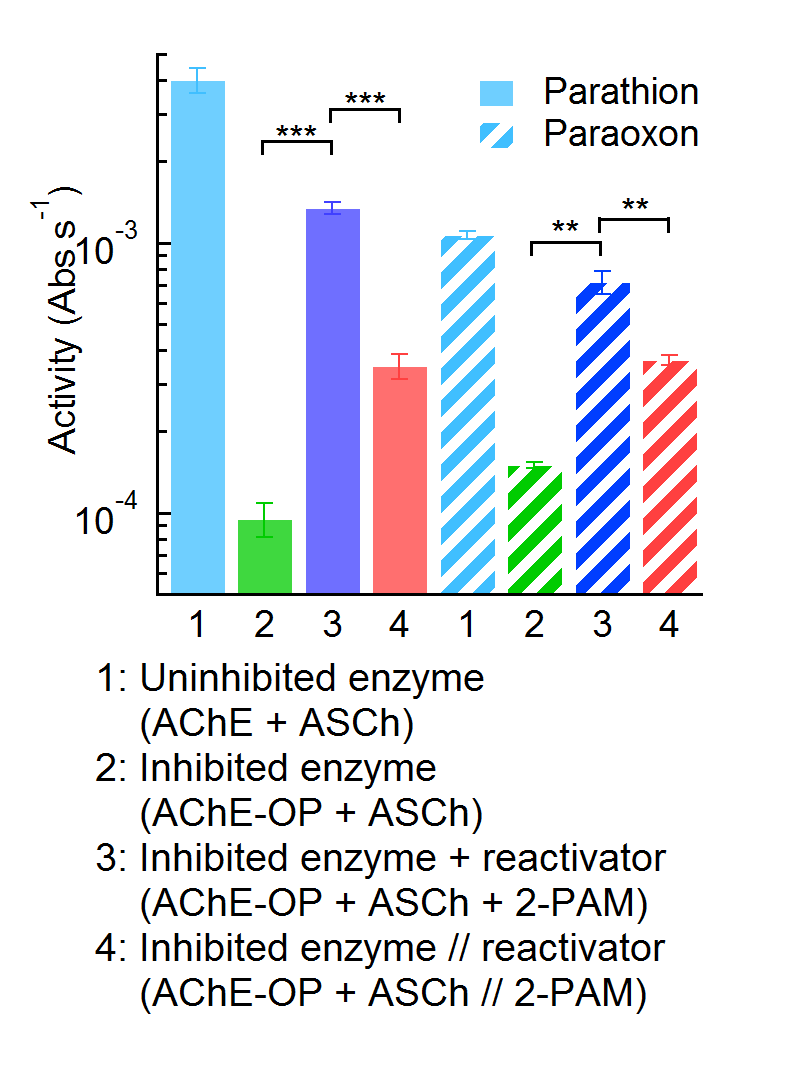


**Figure S1**. Un-normalized AChE activity (dA/dt) obtained from absorbance versus time curves at the inflection point. AChE + ASCh: positive control (uninhibited enzyme + substrate). AChE - OP + ASCh: negative control (inhibited enzyme + substrate). AChE-OP + ASCh + 2-PAM: reactivation with no transport (inhibited enzyme + substrate + reactivator). 2PAM//AChE-OP + ASCh: transcellular transport + reactivation. The differences in activity between uninhibited AChE parathion and paraoxon are due to time-dependent differences in enzyme function.
